# Investigating role of ASIC2 in synaptic and behavioral responses to drugs of abuse

**DOI:** 10.3389/fmolb.2023.1118754

**Published:** 2023-01-30

**Authors:** Margaret J. Fuller, Subhash C. Gupta, Rong Fan, Rebecca J. Taugher-Hebl, Grace Z. Wang, Noah R. R. Andrys, Amal K. Bera, Jason J. Radley, John A. Wemmie

**Affiliations:** ^1^ Department of Psychiatry, University of Iowa, Iowa City, IA, United States; ^2^ Department of Veterans Affairs Medical Center, Iowa City, IA, United States; ^3^ Department of Molecular Physiology and Biophysics, University of Iowa, Iowa City, IA, United States; ^4^ Medical Scientist Training Program, University of Iowa, Iowa City, IA, United States; ^5^ Department of Biotechnology, Bhupat and Jyoti Mehta School of Biosciences, Indian Institute of Technology Madras, Chennai, Tamil Nadu, India; ^6^ Department of Psychological and Brain Sciences, University of Iowa, Iowa City, IA, United States; ^7^ Iowa Neuroscience Institute, University of Iowa, Iowa City, IA, United States; ^8^ Department of Neurosurgery, University of Iowa, Iowa City, IA, United States; ^9^ Interdisciplinary Graduate Program in Neuroscience, University of Iowa, Iowa City, IA, United States

**Keywords:** ASIC, ASIC2, cocaine, opioid, morphine, nucleus accumbens, dendritic spines, acid-sensing ion channel (ASIC)

## Abstract

Drugs of abuse produce rearrangements at glutamatergic synapses thought to contribute to drug-reinforced behaviors. Acid-Sensing Ion Channels (ASICs) have been suggested to oppose these effects, largely due to observations in mice lacking the ASIC1A subunit. However, the ASIC2A and ASIC2B subunits are known to interact with ASIC1A, and their potential roles in drugs of abuse have not yet been investigated. Therefore, we tested the effects of disrupting ASIC2 subunits in mice exposed to drugs of abuse. We found conditioned place preference (CPP) to both cocaine and morphine were increased in *Asic2* ^*−/−*^ mice, which is similar to what was observed in *Asic1a* ^*−/−*^ mice. Because nucleus accumbens core (NAcc) is an important site of ASIC1A action, we examined expression of ASIC2 subunits there. By western blot ASIC2A was readily detected in wild-type mice, while ASIC2B was not, suggesting ASIC2A is the predominant subunit in nucleus accumbens core. An adeno-associated virus vector (AAV) was used to drive recombinant ASIC2A expression in nucleus accumbens core of *Asic2* ^*−/−*^ mice, resulting in near normal protein levels. Moreover, recombinant ASIC2A integrated with endogenous ASIC1A subunits to form functional channels in medium spiny neurons (MSNs). However, unlike ASIC1A, region-restricted restoration of ASIC2A in nucleus accumbens core was not sufficient to affect cocaine or morphine conditioned place preference, suggesting effects of ASIC2 differ from those of ASIC1A. Supporting this contrast, we found that AMPA receptor subunit composition and the ratio of AMPA receptor-mediated current to NMDA receptor-mediated current (AMPAR/NMDAR) were normal in *Asic2* ^*−/−*^ mice and responded to cocaine withdrawal similarly to wild-type animals. However, disruption of ASIC2 significantly altered dendritic spine morphology, and these effects differed from those reported previously in mice lacking ASIC1A. We conclude that ASIC2 plays an important role in drug-reinforced behavior, and that its mechanisms of action may differ from ASIC1A.

## Introduction

Substance use disorders affect millions of people in the United States and worldwide (CDC Health Alert Network, 2020; Krausz et al., 2021; Ahmad FB et al., 2022; National Institute on Drug Abuse, 2022). A better understanding of drug-induced effects on brain function will offer hope for development of new therapies for those afflicted. Multiple drugs of abuse, including cocaine and opioids, induce long-lasting changes at synapses of NAc MSNs which are thought to underlie addiction. These include increased AMPAR/NMDAR ratio, increased GluA2-lacking Ca^2+^-permeable AMPA receptors, and alterations in dendritic spine density and morphology (Lüscher and Malenka, 2011; Thompson et al., 2021; Zinsmaier et al., 2022). Interfering with such drug-evoked synaptic changes in NAc attenuates drug-seeking behaviors in rodents, whereas enhancing synaptic effects of these drugs provokes or exacerbates such behaviors (Kalivas et al., 2009).

Our previous work implicated ASICs in cocaine- and opioid-reinforced synaptic and behavioral effects (Kreple et al., 2014; Gupta et al., 2022). ASICs are cation channels activated by extracellular acidosis. They consist of homo- and heterotrimeric assemblies of channel subunits, primarily ASIC1A, ASIC2A, and ASIC2B (Zha et al., 2006; Zha et al., 2009). ASIC1A is widely expressed in brain neurons. ASIC2A and ASIC2B are alternatively-spliced variants of the *Asic2* gene with different amino-termini (Lingueglia et al., 1997; Zha et al., 2009) and are expressed throughout the brain in a pattern that partially overlaps with ASIC1A (Price et al., 2014; Wu et al., 2016). ASIC1A is a required channel subunit, as disrupting ASIC1A eliminates current evoked by physiological reductions in extracellular pH (from pH 7.4 to pH 5) (Wemmie et al., 2002).

Although ASIC2 subunits are not required for endogenous acid-evoked currents, they do contribute to channel function. Compared to wild-type controls, *Asic2* ^*−/−*^ mice, which lack both ASIC2A and ASIC2B subunits, exhibited significantly reduced acid-evoked current in NAcc MSNs (Kreple et al., 2014). ASIC2A and ASIC2B can assemble with ASIC1A to influence pH sensitivity, channel kinetics, ion selectivity, and sensitivity to modulators including psalmotoxin 1 (PcTx1), Zn^2+^, Ba^2+^, and tetraethylammonium (TEA) (Hesselager et al., 2004; Jiang et al., 2009; Sherwood et al., 2011; Zha, 2013). Although ASIC2A subunits can assemble into homomeric channels, these channels require more extreme pH changes for activation (pH < 5) (Askwith et al., 2004), changes unlikely to be reached in the brain even under pathophysiological conditions. ASIC2B subunits have not been shown to produce functional homomeric channels (Lingueglia et al., 1997).

One endogenous source of acid that may activate these channels *in vivo* is presynaptic neurotransmitter-containing vesicles, which are acidic (pH ∼ 5.5) (Miesenböck et al., 1998) and release protons into the synaptic cleft during neurotransmission (Du et al., 2014). ASIC1A and ASIC2 subunits have been localized to post-synaptic dendritic spines and synaptosomes (Zha et al., 2006; Zha et al., 2009), where they are positioned to respond to synaptically released protons. Consistent with this possibility, ASIC-dependent synaptic currents have been identified during synaptic transmission in a variety of neuron types, including NAcc MSNs (Du et al., 2014; Kreple et al., 2014; González-Inchauspe et al., 2017; Gupta et al., 2022). Inhibiting or genetically disrupting ASIC1A eliminated this current, while disrupting ASIC2 subunits reduced these currents by ∼50% (Kreple et al., 2014), suggesting that both ASIC1A and ASIC2 subunits contribute to the synaptically-evoked currents.

Supporting a role for ASICs in responses to drugs of abuse, disrupting ASIC1A increased CPP to cocaine and morphine (Kreple et al., 2014) and altered acute and chronic locomotor responses to cocaine (Jiang et al., 2013). ASIC1A disruption also increased Ca^2+^-permeable AMPA receptors in NAcc MSNs and AMPAR/NMDAR ratio, an effect which was reversed by acute cocaine exposure. Disrupting ASIC1A also altered synaptic structure, leading to increased dendritic spine density and altered spine characteristics (Kreple et al., 2014).

Roles of the ASIC2 subunits in these responses have not been previously assessed, other than cocaine-evoked locomotor responses (Jiang et al., 2013). We therefore explored consequences of disrupting ASIC2 subunits on cocaine- and morphine-reinforced behavior, as well as synaptic responses to cocaine withdrawal. By comparing these results to our previous studies, we investigated whether disrupting ASIC2 produces the same or different effects as ASIC1A. Because ASIC2 subunits assemble with ASIC1A in the channel complex, we hypothesized that disrupting ASIC2 subunits may resemble ASIC1A disruption.

## Materials and methods

### Mice and drugs

Adult C57Bl6/J mice were bred in-house and kept on a 12 h:12 h light:dark cycle with food and water *ad libitum* in cages of 2-5 animals. All experiments included both male and female mice ranging from 9 to 16 weeks of age. Animals within each experiment were matched for age (< 2 week difference). *Asic2* ^−/−^ mice were generated as described in Price et al. (2000). Briefly, a region of the *Asic2* gene was replaced with a neo cassette, disrupting both *Asic2a* and *Asic2b* splice variants. All procedures involving animals were approved by the University of Iowa Institutional Animal Care and Use Committee. 10 mg/kg cocaine-HCl (Research Triangle Institute International supplied by National Institute on Drug Abuse) or 10 mg/kg morphine (Sigma-Aldrich or Research Triangle Institute International supplied by National Institute on Drug Abuse) were injected i.p., dissolved in sterile saline.

### Conditioned place preference

As previously described (Kreple et al., 2014), animals were allowed to habituate to the experiment room in home cages for at least 30 min before each behavior session. On training days (days 2–4), each animal was placed in a two-chamber CPP apparatus (Med Associates Inc.), and a separation panel was used to keep the two contextually distinct chambers isolated (white side with mesh floor or black side with rod floor). Each animal was injected with drug (10 mg/kg cocaine or 10 mg/kg morphine) or saline and placed in one side of the apparatus for 30 min. Animals were trained to both the drug-paired and the saline-paired side every day, separated by at least 1 h of rest in the vivarium. The context paired with drug was counterbalanced for all groups. On pretest and posttest days (days 1 and 5), animals were placed in the apparatus for 20 min, during which the separation panel was removed, and animals were allowed to explore freely. The amount of time each animal spent in the drug-paired side was recorded *via* automated scoring, and the difference between pretest and posttest was calculated by simple subtraction. No animals spent more than 75% of the pretest time on one side.

### Virus injections

Adeno-Associated Viruses with AAV1 capsids were used to express *eGFP, Asic2a,* and *Asic2b* for all experiments, except when blotting for ASIC1A protein in Figures 2D, E, in which we used AAV2 capsids. Inverted terminal repeats were from AAV2, and all viruses used the cytomegalovirus promoter (CMV). The *eGFP*-expressing virus was produced in the University of Iowa Gene Transfer Vector Core, and the viruses expressing *Asic2a* and *Asic2b* were produced by Genecopoeia (viral titer > 5.0 × 10^12 GC/mL). Mice were injected bilaterally (0.5 µL) in NAcc *via* stereotaxic injection (1.2 mm anterior to bregma, 1.0 mm lateral from midline and 3.9 mm ventral to the pial surface) and allowed to recover for at least 3 weeks before behavioral experimentation or brain harvesting. The *Asic2a* and *Asic2b* viruses were mixed 70:30 with *eGFP* virus to allow for confirmation of targeting after the experiment, as previously described (Coryell et al., 2008). In behavioral assays, animals were sacrificed shortly after testing, and targeting accuracy was validated using the brain atlas Paxinos and Franklin (2019). Animals were excluded from analysis if significant eGFP signal was not detected in NAcc bilaterally. As a consequence, three mice, which had only unilateral eGFP signal, were excluded from each AAV-*Asic2a*-injected *Asic2* ^*−/−*^ group in Figures 3B, C.

### NAcc isolation and Western blotting

Brains were harvested at least 3 weeks after viral injection. 2-mm coronal sections were allowed to partially freeze on dry ice, and NAcc was dissected bilaterally using a circular punch tool (1.2 mm diameter) and placed in chilled lysis buffer (PBS, 1.0% Triton X-100 and Roche cOmplete Mini Protease Inhibitor Cocktail). Tissue homogenization was performed using a syringe and progressively smaller gauge needles until no visible solids remained. Each sample was centrifuged at 5000 RPM (1677 x g) for 10 min at 4°C, and the supernatant was collected. Western blot was performed as previously described (Kreple et al., 2014). Primary antibodies included: rabbit anti-ASIC2 serum (Price et al., 2014) diluted 1:2000, chicken anti-GAPDH 1:10,000 (Millipore, AB2302), and rabbit anti-ASIC1 serum (Wemmie et al., 2002) diluted 1:1000. Secondary antibodies (diluted between 1:10,000-1:20,000) included: IRDye^®^ 800CW donkey anti-rabbit IgG (LI-COR, 925-32213), and IRDye^®^ 680LT donkey anti-chicken IgG (LI-COR, 925-68028). Membranes were imaged with the Odyssey imaging system (LI-COR).

### Slice preparation

Performed as previously described (Kreple et al., 2014) on 8–16 week old mice. 300 µm coronal sections were collected containing NAcc using a Vibrotome (Vibrotome 1000 Plus Sectioning System, Vibratome Co.) in ice-cold slicing buffer (in mM: 225 Sucrose, 26.0 NaHCO_3_, 1.20 KH_2_PO_4_, 1.90 KCl, 10.0 D-glucose, 1.10 CaCl_2_, 2 MgSO_4_) bubbled with 95% O_2_ and 5% CO_2_. Slices were then transferred to 32°C oxygenated artificial cerebrospinal fluid (ACSF) (in mM: 127 NaCl, 26.0 NaHCO_3_, 1.20 KH_2_PO_4_, 1.90 KCl, 10.0 D-glucose, 2.20 CaCl_2_, 2.00 MgSO_4_). After 30 min, slices were held at room temperature for a 30-min recovery.

### Evoked EPSCs

Medium spiny neurons were identified by morphology. Whole-cell recordings were performed using 2.5–4 MΩ pipettes, Axopatch 200 B amplifier (Axon Instruments) and analyzed using Clampfit (Axon software). Any recordings with variance in excess resistance above 20% were excluded from analysis. Internal recording solution was prepared as follows in mM: 125 cesium methanesulfonate, 20.0 CsCl, 10.0 NaCl, 2.00 Mg-ATP, 0.30 Na-GTP, 10.0 HEPES, 0.20 EGTA, 2.50 QX314, pH 7.3 achieved with drops of CsOH. EPSCs were evoked using a bipolar tungsten electrode, and 100 nM PcTx1 was used to block ASIC2A-lacking ASIC channels. For AMPAR/NMDAR ratio calculation, AMPAR-dependent EPSC amplitude was recorded at −70 mV with 100 µM picrotoxin, and NMDAR-dependent EPSC amplitude was recorded using the late component of the EPSC (60 ms after initial evocation) at +50 mV with 100 µM picrotoxin and 20 µM CNQX. A current-voltage curve was created consisting of AMPAR-dependent EPSC recordings at membrane potentials −70, −30, +30, and +50 mV in 100 µM picrotoxin and 100 µM APV (and 0.10 µM Sigma-Aldrich spermine in the internal solution). The current at −70 mV was divided by the current at +50 mV to calculate AMPAR rectification index. Holding voltage was corrected for liquid junction potential (∼8 mV) as in Kreple et al. (2014). Acid-evoked current was assessed in a solution of pH 7.4 by delivering ACSF adjusted to pH 5.6 through a pressurized pipette to the target cell. Rates of desensitization (τ_d_) were calculated by fitting acid-evoked current traces with standard exponential lines of best fit in Clampfit.

### Dendritic spine analysis

Dye application was performed similarly to Gupta et al. (2022) and imaging similarly to Dumitriu et al. (2012). Following 7 days of saline or 7 days of cocaine (10 mg/kg i.p.) in the home cage and 7 days of withdrawal, mice were anesthetized with ketamine/xylazine and transcardially perfused with 1.5% paraformaldehyde (PFA) in sodium phosphate buffer, pH 7.4. Brains were harvested and left in room temperature 1.5% PFA for at least 1 h, then sliced into 120 µm sections *via* Vibratome (Vibratome 1000 Plus Sectioning System, Vibratome Co.). DiI (Invitrogen) was applied as a powder to NAcc under a dissecting microscope using a glass pipette or a small paintbrush. Slices were left on a shaker at 4°C in pH 7.4 sodium phosphate buffer with 0.01% thimerosal (Sigma) for 48 h to allow dye to diffuse. Slices were mounted on slides with Vectashield Hardset mounting medium (Vector Laboratories) and 120 µm SecureSeal Imaging Spacers (Grace Bio-Labs). Imaging was performed on a Leica SP8 Laser Scanning confocal microscope with ×100 oil-immersion objective with a 1.40 numerical aperture. The voxel dimensions for all images were approximately 50 nm × 50 nm × 50 nm. Dendrites were preferentially selected that were long, straight, non-overlapping segments from terminal branches. Dendrite segments within 5.0 µm from a bifurcation or the terminal end were not included in the analysis. On average, dendrites were 57.7 µm away from the soma and 36.9 µm in length. Distance from soma was defined by the most proximal end of each dendrite. The saline-injected wild-type group included 7 mice, using 21 slices, 57 dendritic segments and a total of 6,150 spines. The cocaine-withdrawn wild-type group included 6 mice, using 19 slices, 53 dendritic segments and a total of 5,199 spines. The saline-injected *Asic2* ^*−/−*^ group included 7 mice, using 21 slices, 57 dendritic segments and a total of 6,392 spines. The cocaine-withdrawn *Asic2* ^*−/−*^ group included 8 mice, using 21 slices, 60 dendritic segments and a total of 6,757 spines. Images were deconvolved using automated express deconvolution with Huygens Essential software (Scientific Volume Imaging). Dendritic spine tracing and characterization of deconvolved images was performed using the Neurolucida 360 (MBF Bioscience) user-guided semiautomated procedure. Spines were classified using head diameter, neck diameter and spine length measured in Neurolucida 360 (Rodriguez et al., 2008) employing the same parameters described previously (Kreple et al., 2014). Briefly, spines were classified as thin or mushroom if the head-to-neck diameter ratio was >1.1:1. Within this subdivision, spines with a head diameter >0.35 μm were classified as mushroom, or otherwise classified as thin. Spines with head-to-neck diameter ratios <1.1:1 were also classified as thin if the ratio of spine length-to-neck diameter was >2.5, otherwise they were classified as stubby. All imaging, deconvolution and spine tracing was performed by experimenters blinded to condition. For individual spine characteristics (spine volume, head diameter, neck diameter and spine length), a ROUT outlier test was performed, and outliers were removed. The spines removed from each group ranged from 0.0% to 5.8% of the total.

### Statistics

All graphs express group mean and standard error of the mean. An unpaired two-tailed Student’s *t*-test determined the difference between two groups, unless the variance between groups was unequal, as determined by the F-test. Then, Welch’s correction was used as indicated to account for unequal variance. If the data were not normally distributed, the difference between two groups was determined by the Mann-Whitney test, as was the case for all individual dendritic spine characteristics (spine volume, head diameter, neck diameter, and spine length). A two-way Analysis of Variance (ANOVA) was used to determine the difference between groups larger than two and to determine interactions between independent variables. Following a ROUT outlier test (Q = 1%), Kolmogorov-Smirnov test was used to compare the distributions of two groups. After ANOVA or Kolmogorov-Smirnov test, *t*-tests were used to assess *a priori* hypotheses between two groups. Sex effects were assessed if the number of animals in each group was sufficient to partition the data. Differences were considered significant if the *p*-value was less than 0.05. Statistical analysis was performed using R and GraphPad Prism 9.

## Results

**FIGURE 1:**
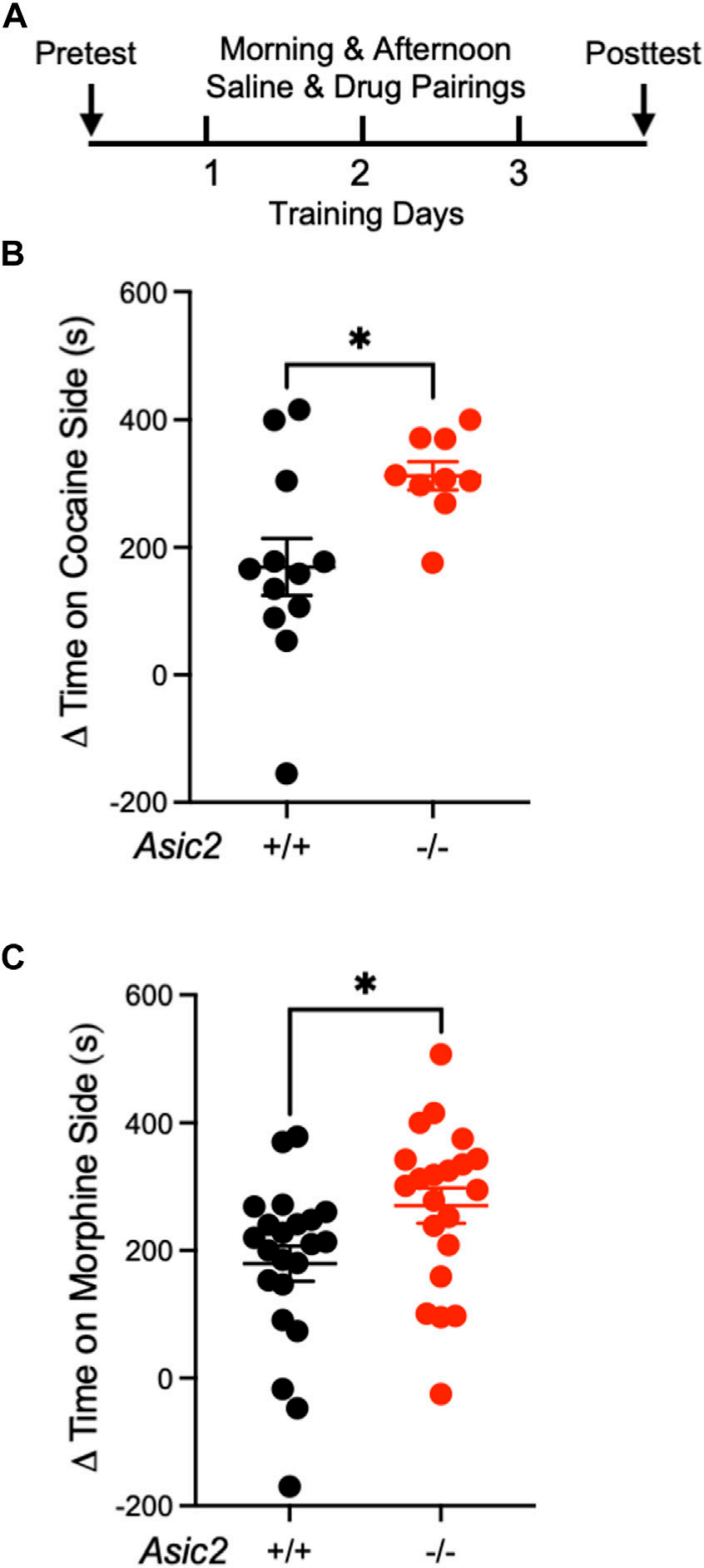
ASIC2 disruption increased conditioned place preference (CPP) to cocaine and morphine. **(A)** Timeline of CPP to cocaine or morphine. **(B)** Difference between posttest and pretest time spent on the cocaine-paired side of the CPP chamber (Δ = simple subtraction) revealed a genotype effect where *Asic2* ^*−/−*^ mice spent more time on the cocaine-paired side [t (15.76) = 2.872, *p* = 0.0112, Welch’s *t*-test, *n* = 12 and 9 mice]. **(C)** Difference between posttest and pretest time spent on the morphine-paired side revealed a genotype effect where *Asic2* ^*−/−*^ mice spent more time on the morphine-paired side [t (41) = 2.326, *p* = 0.0250, Student’s *t*-test, *n* = 22 and 21 mice].

To assess drug-reinforced behavior in *Asic2* ^*−/−*^ mice, we performed CPP to two different drugs of abuse with distinct mechanisms of action, cocaine (10 mg/kg i.p.) and morphine (10 mg/kg i.p.). Because ASIC1A disruption increased CPP to both drugs (Kreple et al., 2014), and because previous work demonstrated shared behavioral phenotypes in *Asic1a* ^*−/−*^ mice and *Asic2* ^*−/−*^ mice (Price et al., 2014), we hypothesized that disrupting ASIC2 subunits would also increase behaviors reinforced by these drugs. After three saline and three cocaine pairings (Figure 1A), both wild-type and *Asic2* ^*−/−*^ mice showed a strong preference for the cocaine-paired side (Figure 1B). Moreover, CPP score was significantly elevated in *Asic2* ^*−/−*^ mice compared to controls. Similar results were observed with CPP to morphine (Figure 1C), where preference for the morphine-paired side was also significantly elevated in the *Asic2* ^*−/−*^ mice relative to wild-types. An interaction effect between sex and genotype was observed in this experiment (Supplementary Figure S1), driven by increased morphine CPP in female wild-type mice versus male wild-type mice. This agrees with previous literature showing elevated opioid-reinforced behavior in female rodents (Becker and Koob, 2016). These results support the hypothesis that ASIC2A, ASIC2B, or both play important roles in drug-reinforced behaviors and raise questions as to whether ASIC2 loss produces these phenotypes by the same or distinct mechanisms as ASIC1A.

**FIGURE 2:**
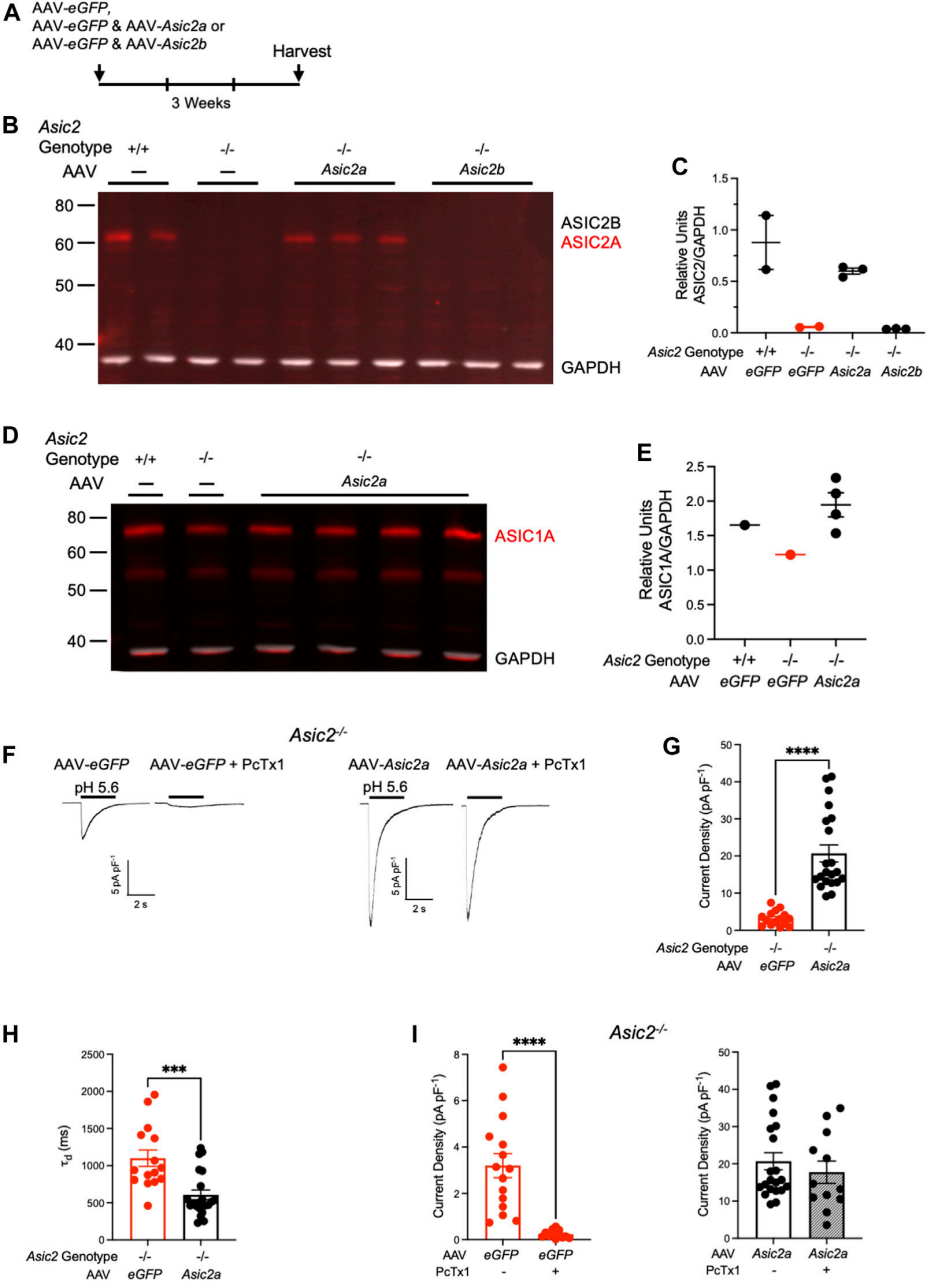
Expressing ASIC2A *via* AAV in NAcc of *Asic2* ^*−/−*^ mice produced functional ASIC2A subunits that integrated with endogenous ASIC1A. **(A)** Timeline of virus injection prior to brain harvesting. **(B)** Western blot of ASIC2 in NAcc isolated from wild-type mice, *Asic2* ^*−/−*^ mice and *Asic2* ^*−/−*^ mice injected with ASIC2A or ASIC2B-expressing virus. Bands for ASIC2 protein are visible in wild-type mice and *Asic2* ^*−/−*^ mice injected with AAV-*Asic2a*. No ASIC2 protein is visible in *Asic2* ^*−/−*^ control or *Asic2* ^*−/−*^ mice injected with AAV-*Asic2b*. **(C)** Quantification of western blot. ASIC2 signal from each lane was normalized to GAPDH signal. **(D)** Western blot of ASIC1A in NAcc isolated from wild-type mouse, *Asic2* ^*−/−*^ mouse and *Asic2* ^*−/−*^ mice injected with ASIC2A-expressing virus. Bands for ASIC1A protein are visible in each condition. **(E)** Quantification of western blot. ASIC1A signal from each lane was normalized to GAPDH signal. **(F)** Representative traces of EPSCs evoked by pH 5.6 in *Asic2* ^*−/−*^ NAcc MSNs transfected with AAV*-eGFP* or AAV*-Asic2a.* Following acid-evoked current, selective ASIC blocker PcTx1 was applied in the same cells. **(G)** Comparing current density in *Asic2* ^*−/−*^ NAcc MSNs reveals a significant effect of AAV-*Asic2a* on acid-evoked current [t (22.01) = 7.389, *p* < 0.0001, Welch’s *t*-test, *n* = 15 and 21 cells]. **(H)** Comparing rates of desensitization (τ_d_) of acid-evoked currents in *Asic2* ^*−/−*^ NAcc MSNs revealed a significant effect of AAV-*Asic2a* compared to controls injected with only AAV-*eGFP* [t (34) = 4.048, *p* = 0.0003, Student’s *t*-test, *n* = 15 and 21 cells]. **(I)** PcTx1 significantly reduced acid-evoked current in *Asic2* ^*−/−*^ MSNs transfected with AAV-*eGFP* (left) [t (14.25) = 5.664, *p* < 0.0001, Welch’s *t*-test, *n* = 15 and 12 cells]. There was no effect of PcTx1 on acid-evoked current in *Asic2* ^*−/−*^ MSNs transfected with AAV*-Asic2a* (right) [t (31) = 0.7707, *p* = 0.4467, Student’s *t*-test, *n* = 21 and 12 cells].

Because NAcc is thought to be an important site of ASIC action in cocaine seeking (Kreple et al., 2014; Gupta et al., 2022), we assessed expression of ASIC2A and ASIC2B subunits at this site. We dissected out NAcc bilaterally from wild-type and *Asic2* ^*−/−*^ mice and assessed ASIC2 expression *via* western blot. Our ASIC2 antibody binds to a C-terminus common to both ASIC2A and ASIC2B subunits (Price et al., 2000). In wild-type mice, a single band was detected in NAcc that was absent in *Asic2* ^*−/−*^ mice (Figure 2B). This band migrated at ∼63 kD, approximately where the ASIC2A subunit has been suggested to migrate (Wu et al., 2016). To test whether this ∼63 kD band contained the ASIC2A or ASIC2B subunit, we transduced the NAcc of *Asic2* ^*−/−*^ mice with AAV virus vectors expressing recombinant ASIC2A or ASIC2B *via* the CMV promoter (Figure 2A). These vectors were identical other than their protein-encoding insert (Genecopoeia, Inc.). We found that the recombinant ASIC2A protein migrated at ∼63 kD, the same molecular weight as the band in wild-type mice. Moreover, recombinant ASIC2A expression in *Asic2* ^*−/−*^ mice was restored to near normal levels (Figures 2B, C). Previous studies using the same antibody suggested the ASIC2B protein migrates at a higher molecular weight (∼75 kD) than ASIC2A (Hruska-Hageman et al., 2002; Wu et al., 2016). Interestingly, we did not detect it in NAcc in wild-type samples. We also did not detect this higher molecular weight band in *Asic2* ^*−/−*^ mice following transduction with the ASIC2B-expressing AAV. Together these results suggest that ASIC2A is the predominant ASIC2 variant in NAcc, and that little or no ASIC2B is present. We further tested ASIC1A subunit expression in NAcc and found that it was not grossly affected by *Asic2* gene disruption or by recombinant ASIC2A expression (Figures 2D, E).

We next assessed whether the virally-expressed ASIC2A subunit was functional by testing acid-evoked current in NAcc MSNs in acute slices. We found that acid-evoked currents were significantly increased in *Asic2* ^*−/−*^ neurons transduced with AAV-*Asic2a* compared to those transduced with AAV-*eGFP* alone (Figures 2F, G). These currents were comparable in magnitude to acid-evoked currents previously reported in wild-type mice (Kreple et al., 2014). Acid-evoked currents decayed more quickly in NAcc MSNs transduced with AAV-*Asic2a* (Figure 2H), which is consistent with a shift from ASIC1A homomeric channels to ASIC1A/ASIC2A heteromers (Askwith et al., 2004). Moreover, the currents in *Asic2* ^*−/−*^ neurons transduced with AAV-*Asic2a* were unaffected by psalmotoxin 1 (PcTx1), which contrasted sharply with the near total block in neurons transduced with AAV-*eGFP* alone (Figure 2I). Because ASIC1A/ASIC2A-containing heteromeric channels are known to be insensitive to PcTx1 in these conditions (Sherwood et al., 2011), results suggest that AAV-*Asic2a* produced functional ASIC2A subunits that heteromultimerized with endogenous ASIC1A.

**FIGURE 3:**
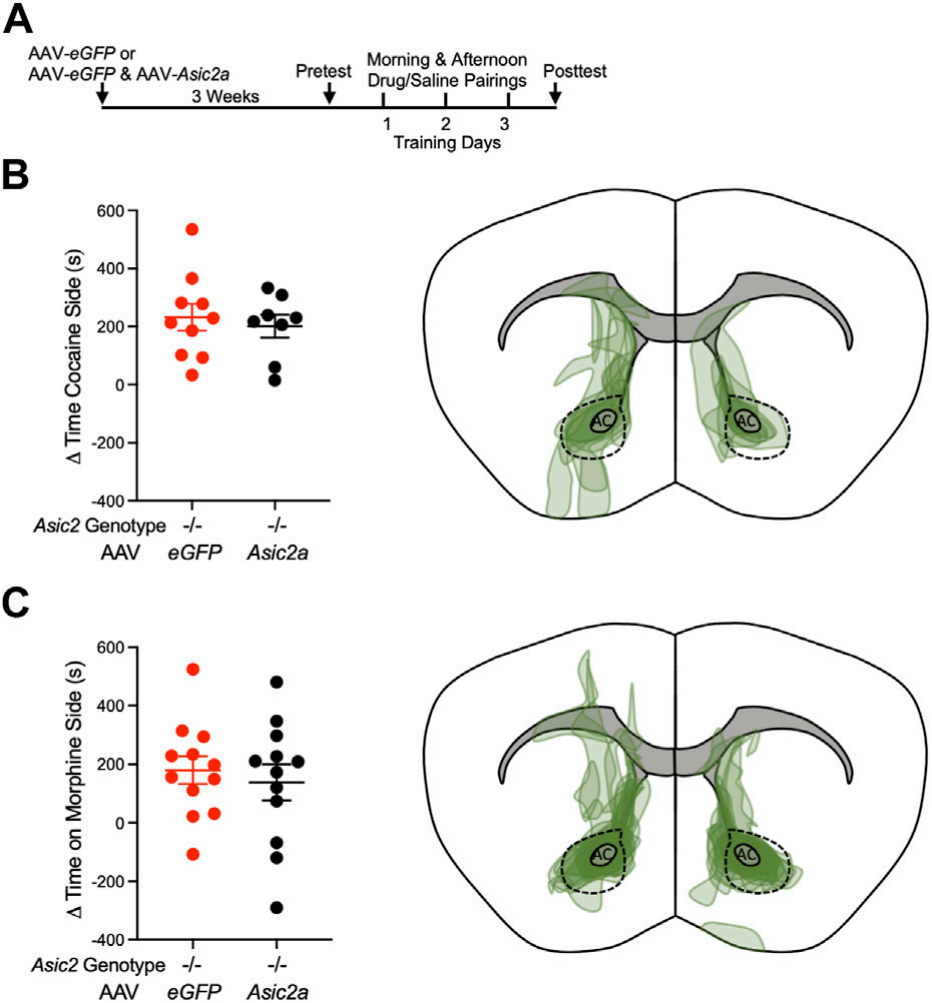
Expressing ASIC2A *via* AAV in NAcc of *Asic2* ^*−/−*^ mice did not change CPP to cocaine or morphine. **(A)** Timeline of virus injection and post-operative recovery prior to CPP to cocaine or morphine. **(B)** Difference between posttest and pretest time on the cocaine-paired side revealed no effect of AAV-*Asic2a* in NAcc of *Asic2* ^*−/−*^ mice [t (16) = 0.4853, *p* = 0.6340, Student’s *t*-test, *n* = 10 and 8 mice]. Targeting map of AAV-*Asic2a* virus in cocaine CPP animals. **(C)** Difference between posttest and pretest time on the morphine-paired side revealed no effect of AAV-*Asic2a* in NAcc of *Asic2* ^*−/−*^ mice [t (22) = 0.5310, *p* = 0.6007, Student’s *t*-test, *n* = 12 mice]. Targeting map of AAV-*Asic2a* virus in morphine CPP animals. AC, anterior commissure; dotted lines encircle NAcc.

The ability to restore ASIC2A protein to near normal levels in NAcc in *Asic2* ^*−/−*^ mice provided an opportunity to test whether NAcc is a behaviorally relevant site of ASIC2A action in cocaine and/or morphine CPP. We transduced the NAcc of *Asic2* ^*−/−*^ mice bilaterally with AAV-*Asic2a* versus AAV-*eGFP* alone. Mice were allowed 3 weeks to recover from the surgery and to express recombinant ASIC2A protein in NAcc (Figure 3A). In two separate cohorts of mice, we tested CPP to cocaine and morphine as before. We hypothesized that restoring ASIC2A in NAcc of *Asic2* ^*−/−*^ mice would reduce CPP, as previously observed with ASIC1A (Kreple et al., 2014). However, contrary to our hypothesis, expressing ASIC2A in NAcc MSNs was not sufficient to alter cocaine CPP (Figure 3B), or morphine CPP (Figure 3C). Thus, although expressing ASIC1A in NAcc was sufficient to reduce the elevated drug-reinforced behavior in *Asic1a* ^*−/−*^ mice (Kreple et al., 2014), expressing ASIC2A in NAcc in *Asic2* ^*−/−*^ mice was not sufficient. These observations suggest that loss of ASIC2 may influence drug-reinforced behaviors differently from loss of ASIC1A.

**FIGURE 4:**
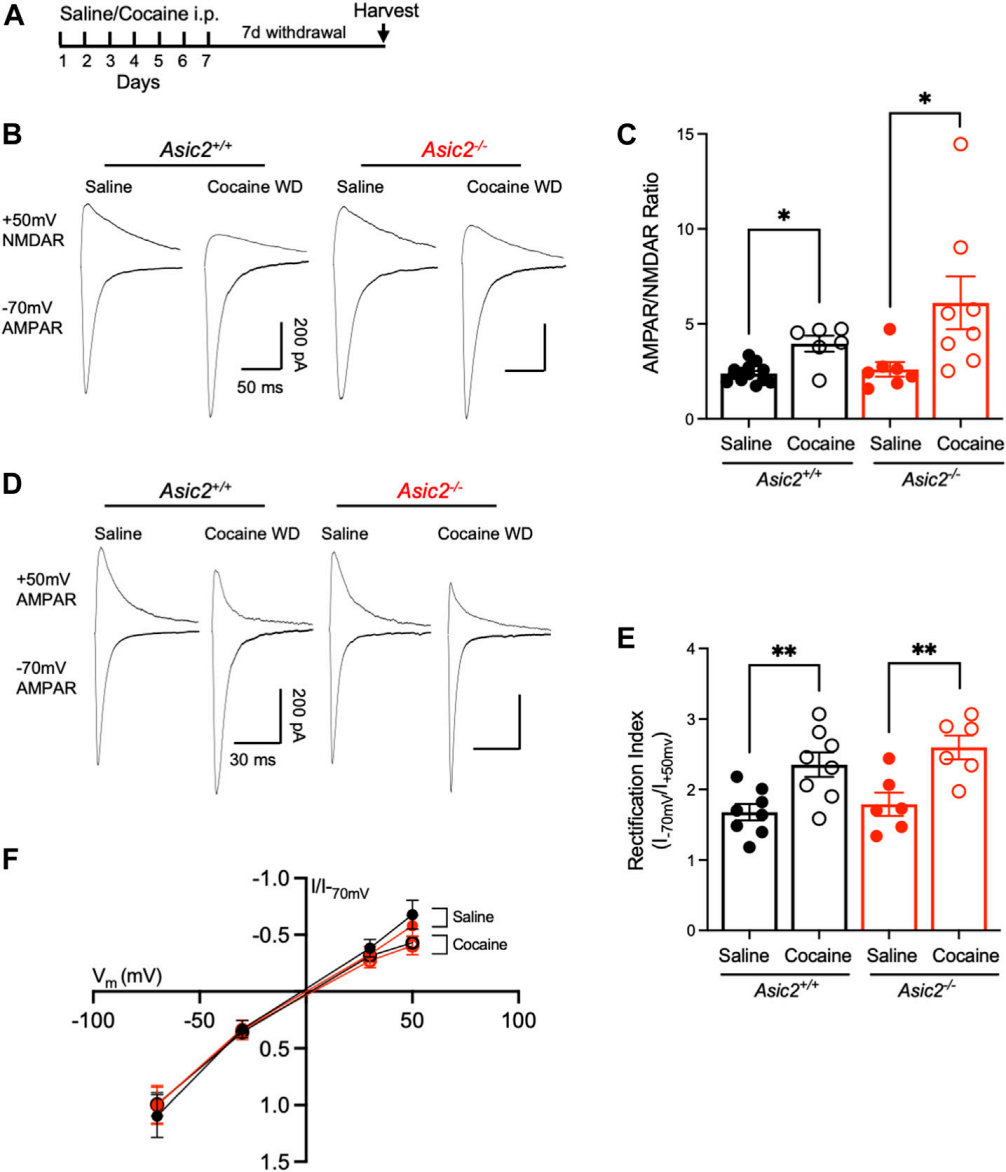
Disrupting ASIC2 subunits did not alter AMPAR/NMDAR ratio or rectification index in NAcc in acute slices at baseline or after cocaine withdrawal. **(A)** Timeline of saline or cocaine home cage injections followed by withdrawal. **(B)** Representative traces of AMPA receptor-mediated EPSC at −70 mV holding potential and NMDA receptor-mediated EPSC at +50 mV holding potential of designated groups. **(C)** An ordinary two-way ANOVA assessing AMPAR/NMDAR ratio revealed a significant effect of cocaine withdrawal but no effect of disrupting ASIC2 and no interaction between genotype and drug exposure [F (1,30) = 12.30, *p* = 0.0014 drug effect; F (1,30) = 2.661, *p* = 0.1133 genotype effect; F (1,30) = 1.786, *p* = 0.1915 interaction, *n* = 6–13 cells]. T-tests revealed an effect of cocaine withdrawal in wild-type [t (5.983) = 3.577, *p* = 0.0117, Welch’s *t*-test, *n* = 13 and 6 cells] and *Asic2* ^*−/−*^ mice [t (8.060) = 2.425, *p* = 0.0413, Welch’s *t*-test, *n* = 7 and 8 cells]. A *t*-test revealed no effect of ASIC2 disruption in cocaine withdrawn groups [t (8.232) = 1.476, *p* = 0.1771, Welch’s *t*-test, *n* = 6 and 8 cells]. **(D)** Representative traces of AMPA receptor-mediated EPSC at −70 and +50 mV holding potential for designated groups. **(E)** An ordinary two-way ANOVA assessing the ratio of AMPA receptor-mediated current at −70 mV to +50 mV revealed a significant effect of cocaine withdrawal on inward rectification but no effect of disrupting ASIC2 and no interaction between genotype and drug exposure [F (1,24) = 22.04, *p* = < 0.0001 drug effect; F (1,24) = 1.293, *p* = 0.2667 genotype effect; F (1,24) = 0.1723, *p* = 0.6818 interaction, *n* = 6-8 cells each]. T-tests revealed an effect of cocaine withdrawal in wild-type (t (14) = 3.240, *p* = 0.0059, Student’s *t*-test, *n* = 8 cells each) and *Asic2* ^*−/−*^ mice [t (10) = 3.422, *p* = 0.0065, Student’s *t*-test, *n* = 6 cells each) **(F)** Current-voltage relationship of AMPA receptor-mediated current in wild-type and *Asic2* ^*−/−*^ NAcc MSNs. WD, withdrawal.

To assess the physiological impact of ASIC2 disruption in NAcc, we quantified several synaptic measures found previously to be altered by ASIC1A disruption and thought to be important in drug-reinforced behaviors. We first tested AMPAR/NMDAR ratio, which has received increasing attention as a marker of cocaine-induced plasticity as it likely plays a critical role in drug-seeking behaviors (Ungless et al., 2001; Kourrich et al., 2007). *Asic2* ^*−/−*^ mice were given cocaine (10 mg/kg i.p.) or saline injections daily in the home cage for 7 days followed by 7 days without injections, during which time the cocaine-injected mice underwent withdrawal. Brains were then harvested for analyses (Figure 4A). Because ASIC2 disruption produced effects similar to ASIC1A disruption on CPP, we hypothesized ASIC2 disruption may produce similar effects on AMPAR/NMDAR ratio (Kreple et al., 2014). However, loss of ASIC2 subunits did not alter baseline AMPAR/NMDAR ratio in saline-injected animals (Figures 4B, C). Moreover, cocaine withdrawal similarly increased AMPAR/NMDAR ratio in both wild-type and *Asic2* ^*−/−*^ mice. These results contrast with effects of ASIC1A disruption, wherein cocaine withdrawal reduced AMPAR/NMDAR ratio from an elevated baseline (Kreple et al., 2014). These results suggest a potentially important physiological difference between the roles of ASIC2 versus ASIC1A.

We next assessed AMPA receptor subunit composition. Cocaine withdrawal increases GluA2-lacking, Ca^2+^-permeable AMPARs (CP-AMPARs) in the postsynaptic membrane which are identifiable by an increase in rectification index (Lüscher and Malenka, 2011). Importantly, *Asic1a* ^*−/−*^ mice were found to exhibit elevated CP-AMPARs at baseline (Kreple et al., 2014). Here we found that cocaine withdrawal significantly increased rectification index in both genotypes, but there were no differences between wild-type and *Asic2* ^*−/−*^ mice (Figures 4D–F). Together with AMPAR/NMDAR ratio, these results suggest that *Asic2* ^*−/−*^ mice have normal synaptic strength and AMPA receptor subunit composition in NAcc MSNs. Furthermore, these results strengthen the possibility that ASIC2 disruption likely influences drug-reinforced behaviors by mechanisms distinct from ASIC1A.

**FIGURE 5:**
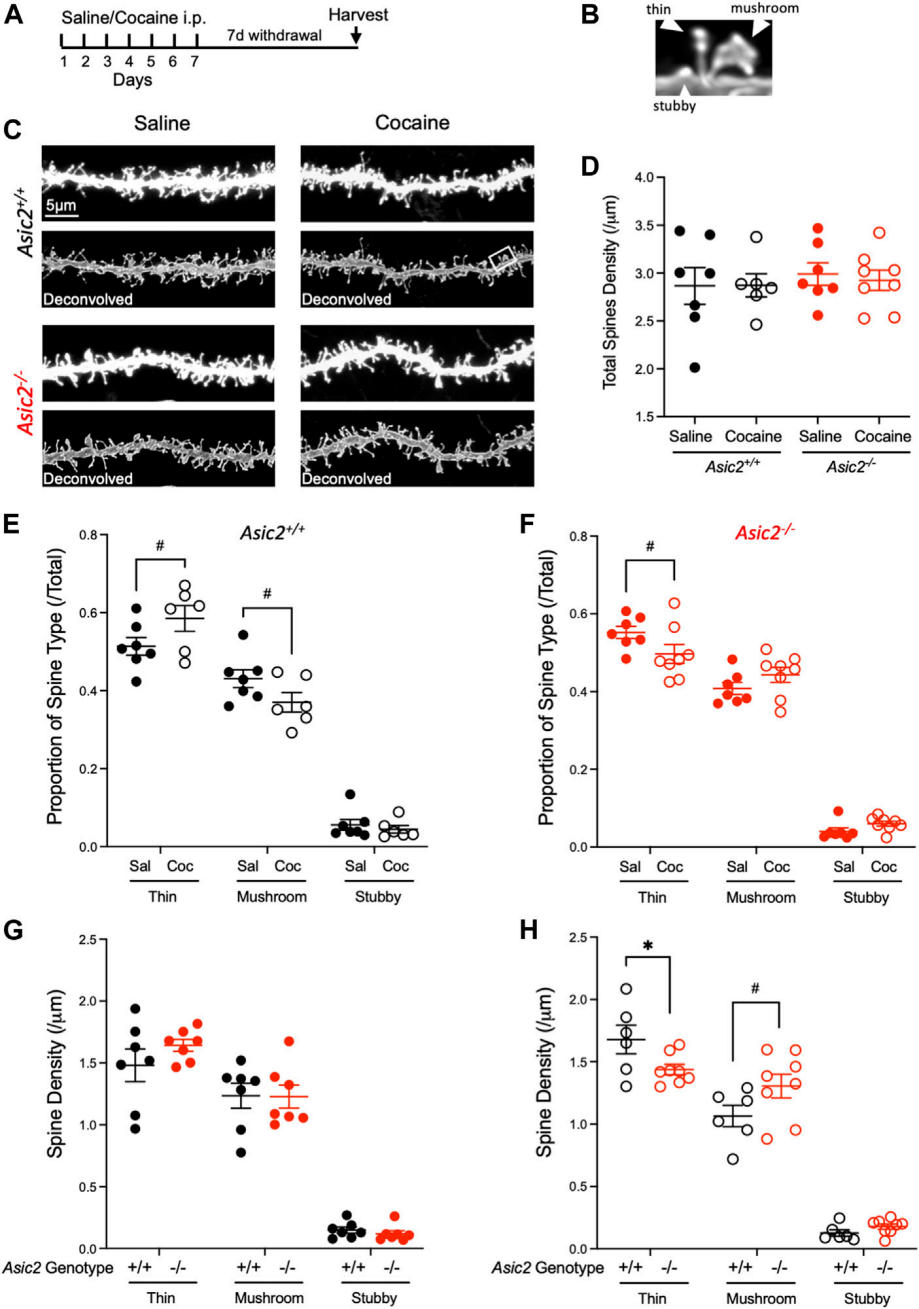
ASIC2 disruption interacted with cocaine withdrawal to produce a shift in spine type that was opposite of withdrawal effects in wild-type mice. **(A)** Timeline of saline or cocaine home cage injections. **(B)** Examples of spine type designations, thin, stubby and mushroom. **(C)** Representative images of NAcc dendrites from wild-type and *Asic2* ^*−/−*^ MSNs after saline injections or cocaine withdrawal before and after deconvolution. The white rectangle shows the spines in **(B)**. **(D)** There was no effect of genotype or drug withdrawal on total dendritic spine density in NAcc *via* two-way ANOVA and no interaction [F (1,24) = 0.4099, *p* = 0.5281 genotype effect; F (1,24) = 0.04724, *p* = 0.8298 drug effect; F (1,24) = 0.06432, *p* = 0.8019 interaction, *n* = 6–8 mice]. **(E)** Proportion of each spine type out of total spine density in wild-type mice. An ordinary two-way ANOVA revealed an interaction between cocaine withdrawal and spine type distribution in wild-type mice [F (2,33) = 4.508, *p* = 0.0186, *n* = 6–7 mice]. Significance bars show *p* values for Student’s t-tests when there was a trend (*p*-value ≤ 0.10, designated #). [t (11) = 1.832, Student’s *t*-test, *p* = 0.0942 thin spines; t (11) = 1.798, Student’s *t*-test, *p* = 0.0997 mushroom spines] **(F)** Proportion of each spine type out of total spine density in *Asic2* ^*−/−*^ mice. An ordinary two-way ANOVA revealed an interaction between cocaine withdrawal and spine type distribution in *Asic2* ^*−/−*^ mice [F (2,39) = 4.267, *p* = 0.0211, *n* = 7–8 mice). Significance bars show *p*-values for Student’s *t*-tests when there was a trend (*p*-value ≤ 0.10, designated #). [t (13) = 1.854, Student’s *t*-test, *p* = 0.0866 thin spines] **(G)** Spine density of each spine type in wild-type and *Asic2* ^*−/−*^ mice after saline injections. An ordinary two-way ANOVA revealed no effect of genotype and no interaction between ASIC2 disruption and spine type. [F (1,36) = 0.3889, *p* = 0.5368, genotype effect; F (2,36) = 0.8229, *p* = 0.4472, interaction] **(H)** Spine density of each spine type in wild-type and *Asic2* ^*−/−*^ mice in cocaine withdrawal. An ordinary two-way ANOVA revealed an interaction between ASIC2 disruption and spine type in cocaine-withdrawn animals [F (2,36) = 5.718, *p* = 0.0070, *n* = 6–8 mice]. Significance bars show *p*-values for Student’s t-tests when there was a trend (*p*-value ≤ 0.10, designated #).; *t*-test between wild-type and *Asic2* ^*−/−*^ thin spines was significant [t (12) = 2.189, *p* = 0.0491, Student’s *t*-test, *n* = 6–8 mice]. Sal, saline; Coc, cocaine.

Because disrupting ASIC1A increased density of dendritic spines in NAcc MSNs (Kreple et al., 2014), we wondered whether loss of ASIC2 would similarly affect dendritic spines. A number of previous studies have also reported effects of cocaine withdrawal on spines in NAcc MSNs, however results of these studies have varied. While some have reported increased dendritic spines in NAcc following cocaine withdrawal (Christian et al., 2017; Gupta et al., 2022), others have reported decreased spine density (Dumitriu et al., 2012). Some have reported effects only in specific populations of MSNs, stratified by presynaptic input or dopamine receptor (D1R/D2R) classification (Lee et al., 2006; Kim et al., 2011; MacAskill et al., 2014; Siemsen et al., 2022). Still others have reported no changes in spine density but rather observed changes in morphological characteristics (Siemsen et al., 2022). Reasons for the differences between studies are not clear but may be due at least in part to different methodologies, drug exposures, and/or spine assessments. Here, we chose a cocaine dose and schedule paralleling our electrophysiological experiments: 7 days of cocaine (10 mg/kg i.p.) or saline, followed by 7 days of withdrawal (Figure 5A). We then harvested brain tissue, labelled dendritic spines in NAcc MSNs with lipophilic dye, and quantified total, thin, mushroom, and stubby spines in wild-type versus *Asic2* ^*−/−*^ mice (Figures 5B, C). We found total spine density did not differ significantly between wild-type and *Asic2* ^*−/−*^ mice (Figure 5D). Total spine density was also not affected by cocaine withdrawal. However, there were significant interactions between relative proportions of spine types and withdrawal, which differed between wild-type and *Asic2* ^*−/−*^ mice. In wild-type mice, cocaine withdrawal shifted spine proportions toward thin spines and away from mushroom spines [two-way ANOVA interaction, F (2,33) = 4.508, *p* = 0.0186] (Figure 5E), which agrees with previous studies suggesting cocaine withdrawal can increase thin spines (Marie et al., 2012; Wang et al., 2013; Dias et al., 2015); while in *Asic2* ^*−/−*^ mice cocaine withdrawal shifted spine proportions toward mushroom spines and away from thin spines [two-way ANOVA interaction, F (2,39) = 4.267, *p* = 0.0211] (Figure 5F). These shifts appear to be primarily related to effects of cocaine withdrawal on spine head diameters (Supplementary Figures 2A–C). Unlike what was previously observed in *Asic1a* ^*−/−*^ mice, where thin and stubby spines tended to be increased, ASIC2 disruption did not affect the densities of these spine types in saline-injected animals (Figure 5G). However, following cocaine withdrawal, there was a significant interaction between genotype and spine type [two-way ANOVA interaction, F (2,36) = 5.718, *p* = 0.0070], whereby thin spines were significantly reduced in *Asic2* ^*−/−*^ mice [t (12) = 2.189, *p* = 0.0491, Student’s *t*-test] (Figure 5H).

**FIGURE 6:**
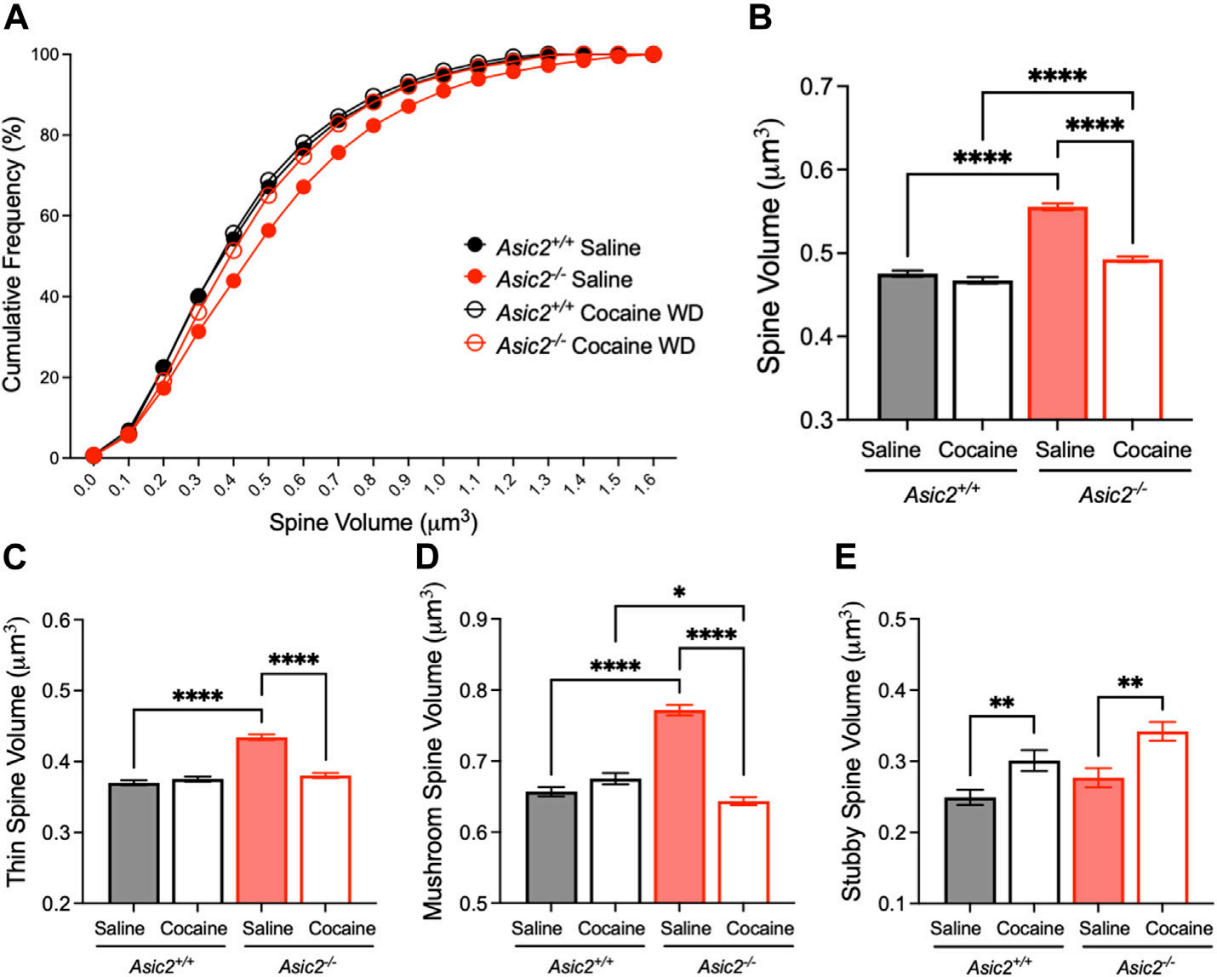
ASIC2 disruption increased overall spine volume, and this effect was reduced by cocaine withdrawal. **(A)** Cumulative distribution of spine volume revealed an overall larger spine size in *Asic2* ^*−/−*^ NAcc MSNs compared to wild-type after saline injections (*p* < 0.0001, Kolmogorov-Smirnov test, *n* = 5893 and 6156 spines) and after cocaine withdrawal (*p* < 0.0001, Kolmogorov-Smirnov test, *n* = 4900 and 6422 spines). There was a significant reduction in spine volume in *Asic2* ^*−/−*^ MSNs following cocaine withdrawal (*p* < 0.0001, Kolmogorov-Smirnov test, *n* = 6156 and 6422 spines). **(B)** Spine volume represented as mean with standard error (*p* < 0.0001, saline *versus* cocaine withdrawn *Asic2* ^*−/−*^; *p* < 0.0001, saline wild-type *versus* *Asic2* ^*−/−*^; *p* < 0.0001, cocaine withdrawn wild-type *versus* *Asic2* ^*−/−*^; Mann-Whitney tests). There was no significant difference between wild-types after saline *versus* cocaine withdrawal (*p* = 0.4314, Mann-Whitney test). **(C)** Volume of thin spines was increased by ASIC2 disruption in saline-injected mice (*p* < 0.0001, Mann-Whitney test, *n* = 3135 and 3446 spines) and decreased by cocaine withdrawal in *Asic2* ^*−/−*^ mice compared to saline (*p* < 0.0001, Mann-Whitney test, *n* = 3446 and 3142 spines). **(D)** Volume of mushroom spines was increased by ASIC2 disruption in saline-injected mice (*p* < 0.0001, Mann-Whitney test, *n* = 2450 and 2491 spines) and decreased by cocaine withdrawal in *Asic2* ^*−/−*^ mice compared to saline (*p* < 0.0001, Mann-Whitney test, *n* = 2491 and 2889 spines). In cocaine withdrawal, *Asic2* ^*−/−*^ mice had reduced mushroom spine volume compared to wild-type (*p* = 0.0345, Mann-Whitney test, *n* = 1832 and 2889 spines). **(E)** Volume of stubby spines was increased in cocaine-withdrawn wild-type mice compared to saline (*p* = 0.0055, Mann-Whitney test, *n* = 313 and 219 spines) and cocaine-withdrawn *Asic2* ^*−/−*^ mice compared to saline (*p* = 0.0034, Mann-Whitney test, *n* = 241 and 390 spines).

Because our data also provided an opportunity to assess spine volume, which has been suggested to be sensitive to cocaine exposure in other brain regions (Radley et al., 2015), we also compared effects of ASIC2 disruption and cocaine withdrawal on this measure. Interestingly, spine volume was significantly increased in saline-injected *Asic2* ^*−/−*^ mice compared to wild-type controls (Figures 6A, B). Moreover, spine volume in *Asic2* ^*−/−*^ mice was highly responsive to cocaine withdrawal, which reduced spine volume back toward normal levels. These effects were observed in both thin and mushroom spines (Figures 6C, D). Spine length was the primary determinant of these volume changes (Supplementary Figures 2F, G). Although stubby spines made up a minority of overall spines, they also showed interesting effects, with cocaine withdrawal increasing stubby spine volume in both genotypes (Figure 6E). Together, these analyses of dendritic spine density and morphology suggest potentially important effects of ASIC2 disruption as well as cocaine withdrawal, raising the possibility that these effects may be related to the behavioral phenotypes in *Asic2* ^*−/−*^ mice. In addition, as with the electrophysiological measures, these effects of ASIC2 disruption appear to differ from those of ASIC1A disruption, although comparable analyses in *Asic1a* ^*−/−*^ mice are not yet available. In particular, neither spine volume nor cocaine withdrawal have been assessed in *Asic1a* ^*−/−*^ mice.

## Discussion

Here we showed that disrupting ASIC2 subunits in mice increased cocaine CPP behavior, as well as opioid CPP, thus suggesting a novel role for ASIC2 subunits opposing drug-reinforced behaviors. Because ASIC2 subunits interact with ASIC1A to form heteromeric channels, we hypothesized that ASIC2 subunits might play a similar role as ASIC1A with a shared mechanism of action. However, in contrast to this expectation, our results identified distinctions between effects of disrupting ASIC2 subunits versus disrupting ASIC1A. Our results suggest that despite shared behavioral phenotypes in CPP, ASIC2 disruption may exert its behavioral effects by different mechanisms than ASIC1A.

Our western blot results suggested that ASIC2A was the predominant ASIC2 subunit in NAcc, which agrees with previous observations in striatum by others (Wu et al., 2016). In wild-type mice, we only detected one band, which migrated at the same molecular weight as recombinant ASIC2A expressed in *Asic2* ^*−/−*^ NAcc. Moreover, the recombinant ASIC2A subunits increased acid-evoked currents in MSNs, which were much less sensitive to PcTx1, suggesting the recombinant ASIC2A integrated with endogenous ASIC1A into functional heteromeric channels. Curiously, we were unable to detect ASIC2B in wild-type NAcc, and in *Asic2* ^*−/−*^ mice transduced with AAV-*Asic2b*, raising the possibility that some endogenous factor(s) may prevent ASIC2B synthesis or accelerate its degradation in NAcc. Previous work suggested that ASIC2B can be sequestered in endoplasmic reticulum (Kweon et al., 2016) where it may be more rapidly degraded. Taken together our results suggest effects of ASIC2 disruption in NAcc are likely due to loss of ASIC2A.

Despite producing functional channels, expressing recombinant ASIC2A in NAcc was not sufficient to affect CPP in *Asic2* ^*−/−*^ mice. In sharp contrast, our previous studies showed that expressing recombinant ASIC1A in NAcc was sufficient to reduce elevated CPP in *Asic1a* ^*−/−*^ mice (Kreple et al., 2014). This apparent contrast suggests there may be a fundamental difference in how these ASIC subunits contribute to their behavioral phenotypes. It would be informative to know whether ASIC2A in NAcc is necessary for normal CPP behavior, like ASIC1A, although region-restricted ASIC2 knockdown would require tools not immediately available to our lab, such as floxed *Asic2* mice. It is conceivable that ASIC2 may act in brain regions different than ASIC1A to affect CPP. Although the expression patterns of ASIC1A and ASIC2 overlap to some degree such as in ventral striatum, cortex and olfactory bulb, there are also differences (Price et al., 2014; Wu et al., 2016). For example, several brain areas appear to express ASIC2 subunits but little or no ASIC1A; these include the interpeduncular nucleus and anterior-medial portion of the bed nucleus of the stria terminalis. Other brain regions appear to have abundant ASIC1A and very little ASIC2, including the lateral habenula, caudate, and putamen (Price et al., 2014). Investigating roles of ASIC2 versus ASIC1A at these different sites may be required to fully understand how these different subunits produce their behavioral effects.

Previous work suggested that disrupting ASIC1A altered AMPAR/NMDAR ratio and AMPA receptor subunit composition in NAcc (Kreple et al., 2014). Our results here indicate neither AMPAR/NMDAR ratio nor AMPAR rectification were altered in NAcc by the loss of ASIC2. Moreover, responses to cocaine withdrawal in these measures did not differ between *Asic2* ^*−/−*^ and wild-type mice. These results highlight several potentially important mechanistic distinctions between effects of ASIC2 versus ASIC1A disruption. Supporting these observations, a previous comparison of cocaine-evoked locomotor responses also suggested differences between *Asic1a* ^*−/−*^ and *Asic2* ^*−/−*^ mice (Jiang et al., 2013).

Further contrasts between ASIC2 and ASIC1A disruption were suggested by our analyses of dendritic spines in NAcc MSNs. Previous work found total spine density was increased in drug-naïve *Asic1a* ^*−/−*^ mice (Kreple et al., 2014), although those studies did not assess effects of cocaine withdrawal. Here, ASIC2 disruption produced no discernible effects on density of total or individual spine types following saline injections. However, we did observe several potentially important interactions between ASIC2 disruption and drug exposure. In wild-type mice, cocaine withdrawal caused a significant shift in distribution of spine types towards thin spines and away from mushroom spines, whereas in *Asic2* ^*−/−*^ mice cocaine withdrawal caused a significant shift towards mushroom spines and away from thin spines. We were further intrigued to find that spine volumes were significantly greater in saline-injected *Asic2* ^*−/−*^ mice compared to wild-type, and that this increase in volume was reversed by cocaine withdrawal. Although more work will be needed to understand how ASIC2 disruption produces these effects on dendritic spines, we speculate that it might be through interactions with intracellular structural proteins. For example, ASIC2 interacts with PICK1 which negatively regulates spine size and binds Rac1, a protein known to alter spine density and cocaine CPP (Hruska-Hageman et al., 2002; Nakamura et al., 2011; Dietz et al., 2012; Rocca and Hanley, 2015).

An important question that remains is how might loss of ASIC2 versus ASIC1A produce different molecular effects if these subunits contribute to the same channel complex. The answer may lie with the different properties that ASIC2 can confer on the heteromeric channel complex compared to ASIC1A homomeric channels. *Via* their association with postsynaptic proteins such as PSD-95, ASIC2 subunits have been suggested to contribute to channel trafficking and localization to synapses (Zha et al., 2009). ASIC2 subunits can also alter sensitivity to synaptic modulators including Zn^2+^ (Chu et al., 2004; Wemmie et al., 2013). ASIC2 subunits modify pH sensitivity, ion permeability, and kinetics including desensitization and recovery from desensitization, which may be critical for some of the biological effects of the channel complex (Hesselager et al., 2004; Sherwood et al., 2011). Finally, differential binding at the C-terminal PDZ domains of ASIC2 versus ASIC1A may lead to different interactions with cytoskeletal architecture. For example, ASIC1A binds alpha actinin, while ASIC2 may not (Schnizler et al., 2009). Conversely, ASIC2 binds PSD-95, while ASIC1A may not (Hruska-Hageman et al., 2004). More work is needed to discern whether any of these properties are critical for the observations herein.

In summary, this paper identifies a novel role for ASIC2 subunits in drug-reinforced behavior and synaptic structure in NAcc MSNs. Our results suggest that ASIC2 subunits achieve their effects through mechanisms distinct from ASIC1A. Future studies will be needed to pinpoint how ASIC2 subunits alter behavior and synaptic structure and function in responses to drugs of abuse. In humans, *ASIC2* gene polymorphisms have been implicated in psychiatric illnesses, including major depressive disorder and panic disorder (Gregersen et al., 2012; Aberg et al., 2018). The present work suggests roles for ASIC2 in humans might also be extended to substance use disorders.

## Data Availability

The original contributions presented in the study are included in the article/Supplementary Materials, further inquiries can be directed to the corresponding author.

## Supplementary material

SUPPLEMENTARY FIGURE S1 An interaction between genotype and sex in morphine CPP was driven by increased morphine CPP in wild-type females. **(A)** Difference between posttest and pretest time spent on the morphine-paired side of the CPP chamber (Δ = simple subtraction) revealed a genotype effect and an interaction between sex and genotype [F (1,39) = 9.906, *p* = 0.0032, genotype effect; F (1,39) = 7.157, *p* = 0.0109, interaction effect] that was driven by a significant increase in behavior in wild-type females compared to wild-type males [t (20) = 2.378, *p* = 0.0275, Student’s *t*-test, *n* = 9 male and 13 female mice]. There was no significant difference between males and females in the *Asic2* ^*−/−*^ groups [t (19) = 1.504, *p* = 0.1489, Student’s *t*-test, *n* = 5 male and 16 female mice].

SUPPLEMENTARY FIGURE S2 ASIC2 disruption and cocaine withdrawal had effects on individual spine morphology measurements. **(A)** Schematic showing each spine morphology measurement, head diameter, neck diameter and spine length. **(B)** Cumulative distributions revealed a decrease in spine head diameter in cocaine-withdrawn wild-type mice versus saline (*p* = 0.0001, Kolmogorov-Smirnov test, *n* = 6049 and 5033 spines) and an increase in cocaine withdrawn *Asic2* ^*−/−*^ mice versus saline (*p* < 0.0001, Kolmogorov-Smirnov test, *n* = 6242 and 6583 spines). In the cocaine withdrawn groups, there was also a significant increase in head diameter in *Asic2* ^*−/−*^ mice versus wild-type (*p* < 0.0001, Kolmogorov-Smirnov test, *n* = 5033 and 6583 spines). **(C)** Spine head diameter represented as mean with standard error [t (10876) = 5.928, *p* < 0.0001, saline versus cocaine withdrawn wild-type; t (12821) = 6.674, *p* < 0.0001, saline versus cocaine withdrawn *Asic2* ^*−/−*^; t (11288) = 12.15, *p* < 0.0001, cocaine withdrawn wild-type versus *Asic2* ^*−/−*^; Welch’s *t*-tests]. **(D)** Cumulative distributions revealed that in *Asic2* ^*−/−*^ mice, there was a significant increase in spine neck diameter distribution in cocaine withdrawal versus saline (*p* = 0.0139, Kolmogorov-Smirnov test, *n* = 6308 and 6618 spines). There was also an increase in spine neck diameter in saline-injected wild-type versus *Asic2* ^*−/−*^ mice (*p* = 0.0125, Kolmogorov-Smirnov test, *n* = 6089 and 6308 spines) and in cocaine withdrawal (*p* < 0.0001, Kolmogorov-Smirnov test, *n* = 5115 and 6618 spines). **(E)** Spine neck diameter represented as mean with standard error. Neck diameter was significantly different between wild-type versus *Asic2* ^*−/−*^ mice after saline injections [t (12387) = 3.195, p = 0.0014, Welch’s *t*-test] and in cocaine withdrawal [t (11249) = 6.269, *p* < 0.0001, Welch’s *t*-test]. **(F)** Cumulative distributions revealed a decrease in spine length in wild-type after saline versus cocaine withdrawal (*p* = 0.0156, Kolmogorov-Smirnov test, *n* = 6209 and 5195 spines) and *Asic2* ^*−/−*^ mice after saline versus cocaine withdrawal (*p* < 0.0001, Kolmogorov-Smirnov test, *n* = 6392 and 6765 spines). There was a significant increase in spine length in *Asic2* ^*−/−*^ mice versus wild-type after saline injections (*p* < 0.0001, Kolmogorov-Smirnov test, n = 6209 and 6392 spines) and in withdrawal (*p* = 0.0248, Kolmogorov-Smirnov test, *n* = 5195 and 6765 spines). **(G)** Spine length represented as mean with standard error. [t (11219) = 2.111, *p* = 0.0348, saline versus cocaine withdrawn wild-type, Welch’s *t*-test; t (13030) = 9.800, *p* < 0.0001, saline versus cocaine withdrawn *Asic2* ^*-/-*^, Welch’s *t*-test; t (12599) = 8.927, *p* < 0.0001, saline-injected wild-type versus *Asic2* ^*−/−*^, Student’s *t*-test; t (11958) = 1.845, *p* = 0.0651, cocaine withdrawn wild-type versus *Asic2* ^*−/−*^, Student’s *t*-test, designated #].

## Abbreviations

ASIC, Acid-Sensing Ion Channels; NAcc, nucleus accumbens core; MSN, medium spiny neuron; CPP, conditioned place preference; i.p., intraperitoneal; AAV, adeno-associated virus; CMV, cytomegalovirus; eGFP, enhanced green fluorescent protein; AMPA, α-amino-3-hydroxy-5-methyl-4-isoxazolepropionic acid; CP-AMPAR, calcium-permeable α-amino-3-hydroxy-5-methyl-4-isoxazolepropionic acid receptor; NMDA, N-methyl-d-aspartate receptor; PcTx1, psalmotoxin 1; PSC, excitatory postsynaptic current; CNQX, 6-cyano-7-nitroquinoxaline-2,3-dione; APV, (2R)-amino-5-phosphonopentanoate; DiI, 1,1′-dioctadecyl-3,3,3′,3′-tetramethylindocarbocyanine perchlorate; PFA, paraformaldehyde.
