# Supplementary figures and images for: Investigating role of ASIC2 in synaptic and behavioral responses to drugs of abuse
Source: Front Mol Biosci. 2023 Jan 30;10:1118754. doi: 10.3389/fmolb.2023.1118754 (PMC9923001; doi:10.3389/fmolb.2023.1118754)

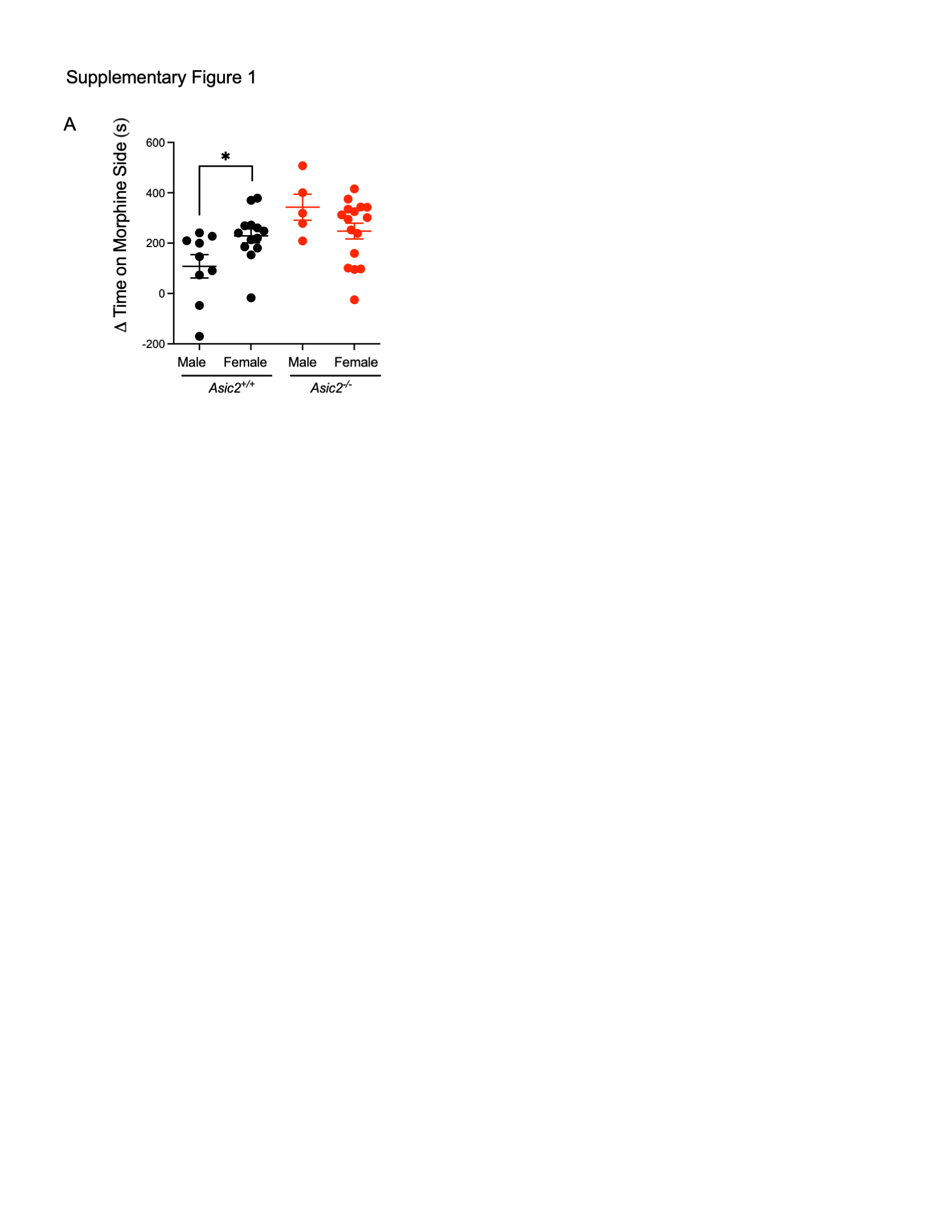

Supplement: Supplementary file 1 [file Image1.tiff]

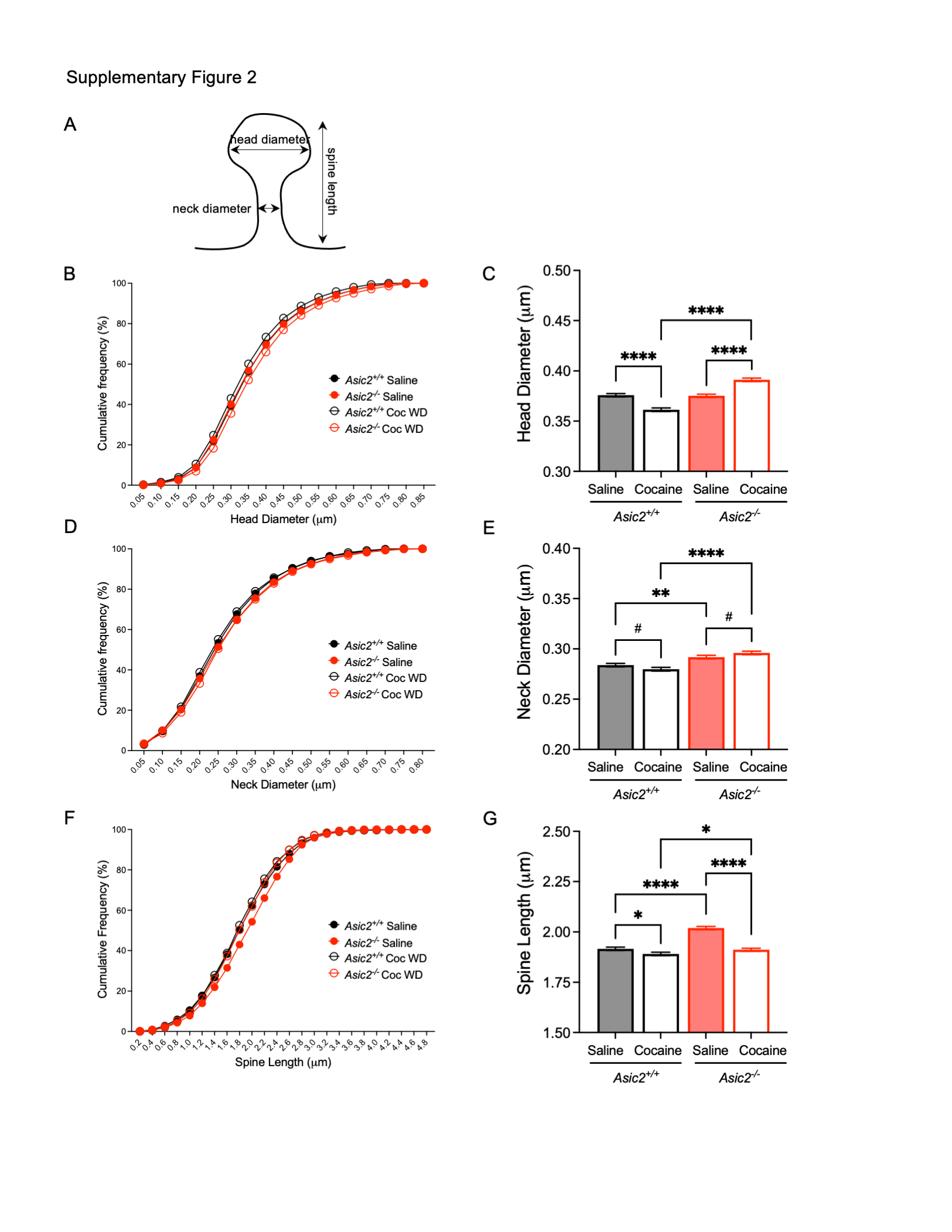

Supplement: Supplementary file 2 [file Image2.tiff]
